# Cyberlogic-enabled Thinking Space: a closed-loop human-cyber cognitive architecture in CPST

**DOI:** 10.3389/frai.2026.1899109

**Published:** 2026-10-28

**Authors:** Ruizi Chen, Lei Zhou, Jianguo Ding, Huansheng Ning

**Affiliations:** 1 School of Computer & Communication Engineering, University of Science and Technology Beijing, Beijing, China; 2 Department of Computer Science, Blekinge Institute of Technology, Karlskrona, Sweden

**Keywords:** closed-loop cognition, cognitive augmentation, cyberlogic, Cyberlogic-enabled Thinking Space (CyTS), Cyber-Physical-Social-Thinking (CPST), human–cyber collaboration

## Abstract

Human–AI collaboration in Cyber–Physical–Social–Thinking (CPST) environments requires coordination among perception, reasoning, human judgment, and governance. This paper proposes Cyberlogic-enabled Thinking Space (CyTS), a conceptual governance-centric architecture organized through Entity, Mapping, Reasoning, and Governance layers. Its closed loop connects multimodal representation, heterogeneous reasoning, collaborative decisions, and controlled adaptation. The Entity layer provides operational descriptions rather than a sufficient ontology of cognition; logical consistency and predictive accuracy do not by themselves establish the causal validity of an action. CyTS therefore distinguishes associative, deductive, interventional, and exploratory claims and requires explicit assumptions, evidence provenance, uncertainty, and verification status at component interfaces. Structural causal models provide one implementable route, while equilibrium-based causal logic and proposals concerning brain–universe similarity and entangled machine thinking are examined as research perspectives with separate validation requirements. Governance mechanisms support privacy, accountability, human oversight, and runtime monitoring, including abstention when evidence or constraints are insufficient. Research hypotheses and evaluation protocols address causal transparency, decision quality, adaptation, cross-space coordination, and energy cost. Healthcare, education, and transportation scenarios illustrate the proposed workflow; they do not constitute empirical validation. CyTS contributes an auditable organizational framework and a testable implementation agenda, without claiming a completed theory of cognition or demonstrated performance gains.

## 1 Introduction

Advances in artificial intelligence, multimodal learning (Roy et al., 2019), brain–computer interfaces (Lorach et al., 2023; Wilson et al., 2025), and ubiquitous sensing are transforming human–machine interaction from tool-based automation toward cognitive collaboration and joint decision-making (Flesher et al., 2021; Wang et al., 2025). Large foundation models provide strong semantic understanding (Vaswani et al., 2017), knowledge organization, and reasoning capabilities, while behavioral and physiological sensing enables partial modeling of human cognitive states. Consequently, AI is evolving from an execution tool into an active participant in perception, reasoning, and decision support.

Despite these advances, reliable integration of reasoning, feedback, and accountability remains a practical challenge (Vaccaro et al., 2024). Human participation and interpretability (Reverberi et al., 2022; Yang et al., 2023) can be incorporated at multiple stages, but their presence alone does not establish that a decision is causally justified or that a physical action is safe. CPST systems therefore need explicit connections among evidence, model assumptions, decision authority, and observed outcomes, particularly when representations and actions cross domain boundaries.

These limitations become more pronounced in Cyber–Physical–Social–Thinking (CPST) environments (Ning and Liu, 2015), where cognition emerges from interactions among physical, social, cyber, and thinking spaces. Although cyber–physical and cyber–social systems have been extensively studied, cyber-enabled thinking space still lacks a systematic organizational architecture for trustworthy human–cyber cognition.

To address this gap, this paper introduces Cyberlogic as a structured organizational framework integrating perception, representation, reasoning, governance, and feedback into a unified cognitive process. Based on Cyberlogic, we further propose Cyberlogic-enabled Thinking Space (CyTS), a governance-centric human–cyber cognitive architecture for CPST environments. CyTS supports cognitive augmentation by coordinating human cognition, cyber intelligence, and governance within a closed-loop process while preserving human agency, interpretability, accountability, and adaptive feedback.

The central research question is how a governance architecture can connect heterogeneous reasoning to accountable action without confusing a coherent representation with a validated causal explanation. Two subsidiary questions concern how components should expose their assumptions and how improvements should be tested against their computational cost. We use “causal-logical transparency” to mean an inspectable connection between a claim, its assumptions, inference steps, and intervention-relevant evidence, rather than the fluency of a generated explanation. CyTS is proposed as an architectural specification; the present study does not report an implemented system or controlled experiments.

The main contributions of this paper are summarized as follows:

1. A governance-centric cognitive architecture, CyTS, is proposed for CPST environments, integrating perception, representation, reasoning, human participation, and feedback into a unified closed-loop process;
2. A four-layer Entity-Mapping-Reasoning-Governance structure is introduced to operationalize Cyberlogic and support coordination between human cognition and cyber intelligence;
3. Governance is embedded directly into the reasoning process through privacy protection, accountability management, human oversight, responsibility allocation, and runtime monitoring mechanisms;
4. Research hypotheses, validation criteria, and operational workflows are provided to improve the testability and implementability of the proposed framework;
5. Cross-domain mappings in healthcare, education, and transportation demonstrate the architectural consistency and potential applicability of CyTS.

Section 2 reviews related architectures and the epistemic limits of their reasoning mechanisms. Section 3 defines CyTS and its hypotheses. Sections 4 and 5 specify the architecture and governance mechanisms. Section 6 presents operational workflows and domain illustrations. Section 7 sets out evaluation requirements and limitations, and Section 8 concludes the paper.

## 2 Related work and structural limitations

### 2.1 Cognitive architectures, distributed cognition, and hybrid intelligence

Cognitive architectures provide models of learning, reasoning, and computational cognition, with particular emphasis on the organization of individual agents (Ning, 2016; Zeng et al., 2020). Distributed cognition broadens the unit of analysis to interactions among people, tools, artifacts, and environments, while hybrid intelligence emphasizes complementary human and machine capabilities (Glickman et al., 2025; Ning et al., 2017). Together, these perspectives motivate the integration of human judgment and cyber computation in CPST environments.

For CyTS, the relevant challenge is to translate these complementary perspectives into explicit cross-space interfaces. An account of cognitive organization or task complementarity does not by itself specify how uncertain observations, reasoning assumptions, human authority, and feedback should be connected in an executable workflow. CyTS builds on these perspectives by proposing an organizational structure for this coordination; it does not claim to replace established cognitive theories or to establish a complete theory of human thinking.

### 2.2 Human–AI governance and cross-space integration

Human-centered AI emphasizes augmentation, reliability, transparency, and human control. Human-in-the-Loop AI, Human–AI Teaming, Interactive Machine Learning, and Mixed-Initiative Systems operationalize these objectives through feedback, task coordination, and shared decision-making. Work on human-like learning and reasoning provides a complementary cognitive perspective (Lake et al., 2017). Causal reasoning (Pearl, 2018), cyberentity modeling (Dhelim et al., 2020), and explainable AI (Pahud de Mortanges et al., 2024) address related requirements for representing and interpreting decisions. Non-invasive BCIs can supply additional cognitive inputs (Edelman et al., 2025), while bounded rationality and free-energy accounts inform models of cognition and adaptation (Kahneman, 2003; Friston, 2010); none of these contributions alone specifies an end-to-end governance architecture.

Governance-oriented approaches address different parts of this integration problem. Constitutional AI uses principles to regulate model behavior; trustworthy AI frameworks articulate lifecycle requirements; and runtime assurance monitors operation and supports intervention within specified conditions (Schierman et al., 2020). These approaches overlap, and particular implementations may already embed constraints and feedback within inference. Governance integration alone therefore does not establish CyTS's novelty or superiority. Its proposed contribution is the explicit connection among CPST representations, reasoning evidence, human authority, and controlled adaptation. Table 1 compares architectural emphases rather than experimentally measured capabilities; the CyTS column states intended functions.

**Table 1:** Architectural emphases and the proposed contribution of CyTS.

Approach	Primary emphasis	Relationship to CyTS
Human-centered/human-in-the-Loop AI	Human control and feedback across design and operation	Supplies oversight methods; CyTS specifies cross-space evidence and decision interfaces.
Human–AI teaming/hybrid Intelligence	Complementary capabilities, coordination, and task allocation	Supplies collaboration methods; CyTS links allocation to governance records.
Constitutional AI	Principles governing model behavior	Supplies policy constraints; CyTS additionally coordinates physical evidence and action review.
Runtime assurance	Monitoring and intervention within a defined operating envelope	Supplies safety mechanisms; CyTS links them to reasoning assumptions and human escalation.
Cognitive digital twins	Representation and state synchronization	Supplies state models; CyTS additionally specifies evidence status and decision authority.
CyTS	CPST-wide coordination of representation, reasoning, governance, and feedback	Intended integration; comparative benefits require implementation and controlled evaluation.

This comparison yields four integration requirements: governance must be connected to intermediate inference, feedback must preserve decision provenance, representations must retain temporal and contextual information, and cross-space actions must remain subject to authority and safety constraints. These are implementation-dependent requirements, not universal deficiencies of existing architectures. Additional monitoring and verification may be justified for consequential decisions but can impose unnecessary overhead in routine, low-risk tasks. Beyond organizational integration, CyTS must also distinguish logically valid statements from empirically supported intervention claims. The following subsections examine this epistemic boundary, alternative causal formalisms, and exploratory perspectives on machine cognition.

### 2.3 Logical validity, causal evidence, and computational cost

An entity-centered description identifies objects and attributes, while truth-valued inference establishes consequences relative to premises. Neither alone supplies a model of how an intervention changes a physical or social process. This limitation does not imply that symbolic methods are intrinsically unsuitable for AI: explicit rules remain useful for checking consistency, permissions, and constraints. The missing step is the connection between formal symbols, measured processes, and action-dependent outcomes. Likewise, a probabilistic predictor estimates regularities under its data-generating conditions; predictive success need not transfer when an action changes those conditions.

LLMs can assist with causal hypotheses and formalization, but generated reasoning is not automatically a faithful account of the computation that produced an answer. Experiments demonstrate misleading chain-of-thought explanations (Turpin et al., 2023), and CLadder separates associational, interventional, and counterfactual questions to test formal causal reasoning (Jin et al., 2023). These results motivate independent checks of intermediate claims, rather than a blanket assertion that LLMs cannot reason. In CyTS, an explanation must distinguish retrieved evidence, model assumptions, deductive consequences, and unverified suggestions.

Causal opacity also complicates the diagnosis of unnecessary generation, repeated calls, and failed action proposals. However, opacity is not an established universal cause of high energy consumption. Deployment measurements show that energy depends on task and model configuration (Luccioni et al., 2024); adding verification can itself increase cost. We therefore treat any efficiency benefit of transparent reasoning as a hypothesis to test at matched decision quality and safety.

### 2.4 Causality beyond truth-valued representation

Pearl's (2018) structural causal framework distinguishes observation from intervention and counterfactual inference. It is not merely a restatement of empirical correlation or Boolean truth. Its conclusions are conditional on structural assumptions, intervention semantics, and identification conditions; data alone do not generally determine those assumptions. Thus, CyTS must expose possible confounding, model misspecification, and the limits of transporting a causal claim across environments.

An alternative line of work seeks logically definable causality through equilibrium-based regularity. Zhang and Peace (2014) propose bipolar dynamic logic (BDL) and bipolar quantum linear algebra (BQLA), framing causation through bipolar interactions rather than only truth-valued descriptions of beings. Their account is positioned against the limitations they identify in Aristotelian and Humean treatments of causation. Here, “formal causality” denotes derivability within a specified formal system; it is not used as a synonym for Aristotle's formal cause. This perspective broadens CyTS's candidate reasoning formalisms, but a derivation in BDL does not by itself establish that an encoded CPST mechanism is empirically correct.

Equilibrium also appears within conventional dynamical causal modeling. Mooij et al. (2013) establish conditions under which equilibria of differential equations admit structural causal representations. This connection should not be conflated with BDL's broader foundational claims. In either approach, CyTS requires a specified state space, interaction rules, intervention semantics, and conditions for existence, uniqueness, and stability where relevant. An observed balance or a fitted fixed point alone is insufficient evidence of a causal mechanism.

### 2.5 Brain–universe similarity and exploratory quantum perspectives

Vazza and Feletti (2020) report quantitative similarities between selected neuronal-network and cosmic-web structural measures, using proximity-based approximations of neural connectivity. Such observations can motivate hypotheses about network organization; they do not establish shared cognitive functions or a common causal mechanism. CyTS therefore treats structural analogy as a source of candidate representations, not as evidence that a cosmic or quantum model explains human thinking.

Zhang (2024) proposes global realism with bipolar strings, a causal-logical account of quantum gravity, and causal-logical brain modeling for entangled machine thinking and imagination (EMTI), including a proposed mind–light–matter unity. These proposals connect foundational physics, quantum information science, and machine cognition. For CyTS, they motivate exploratory relational reasoning modules. Their adoption would require an executable specification and discriminating tests beyond the architectural illustrations presented here. Computational coupling of representations must also be distinguished from experimentally demonstrated physical quantum entanglement. CyTS neither assumes a quantum substrate for cognition nor claims to validate quantum gravity.

## 3 Cyberlogic-oriented structural foundation of CyTS

### 3.1 Definition and organizational role of Cyberlogic

Cyberlogic is a foundational framework proposed for organizing cross-space reasoning, logical coordination, and governance within the Cyber-Physical-Social-Thinking (CPST) hyperspace (Ning et al., 2023, 2017). Unlike conventional AI reasoning frameworks that primarily focus on inference efficiency or model performance, Cyberlogic emphasizes the structural organization, traceability, semantic consistency, and governance of reasoning processes across heterogeneous spaces (Durstewitz et al., 2019). Within the Cyberlogic framework, logical activities are organized through four interconnected layers:

- **Entity layer:** the Entity layer defines the cyberentities participating in reasoning processes, including physical entities, social entities, cognitive entities, and cyber-enabled entities. Cyberlogic treats entities as structured logical carriers with unified attribute schemas supporting identity, spatiotemporal states, social semantics, cognitive states, and security constraints.
- **Mapping layer:** the Mapping layer transforms entities, relationships, and cognitive states from physical, social, and thinking spaces into cyber representations capable of participating in logical operations. Cyberlogic explicitly emphasizes that mapping is not equivalent replication, but selective reconstruction with uncertainty and semantic abstraction.
- **Reasoning layer:** the Reasoning layer coordinates multiple logic sources during inference, including both cyberized logic migrated from PST spaces and endogenous cyberlogic generated within cyberspace through data-driven and generative mechanisms. Rather than unifying all logic into a single reasoning formalism, Cyberlogic focuses on multi-logic coordination, reasoning traceability, uncertainty propagation, and conflict resolution.
- **Governance layer:** the Governance layer provides meta-level regulation for the entire reasoning process. It ensures semantic consistency, traceability, integrity maintenance, responsibility allocation, and controllable evolution of reasoning systems through governance constraints and operational principles (Mittelstadt et al., 2016).

The Entity layer is an engineering device for identifying participants, observations, and responsibilities; it does not commit CyTS to a being-qua-being account in which an ontology of entities suffices to explain cognition. Processes, events, interventions, and time-dependent relations must be represented alongside entities. Similarly, “multi-logic” denotes coordination among explicitly specified methods, not a new universal calculus. A method may establish formal validity within its assumptions while leaving empirical adequacy unresolved. Governance must retain that distinction when authorizing actions.

Within this theoretical foundation, CyTS can be understood as a Cyberlogic-oriented cognitive architecture specifically focused on governance-aware augmentation of the Thinking Space in CPST environments. Rather than replacing human cognition, CyTS extends Cyberlogic toward recursive human–cyber collaborative cognition, adaptive reasoning coordination, and cognitive governance under multi-space interaction conditions. To clarify the theoretical position of CyTS within CPST, Figure 1 illustrates how Cyberlogic functions as the organizational logic that connects physical inputs, social contexts, cyber models, and human cognition into a closed-loop cognitive structure.

**Figure 1:**
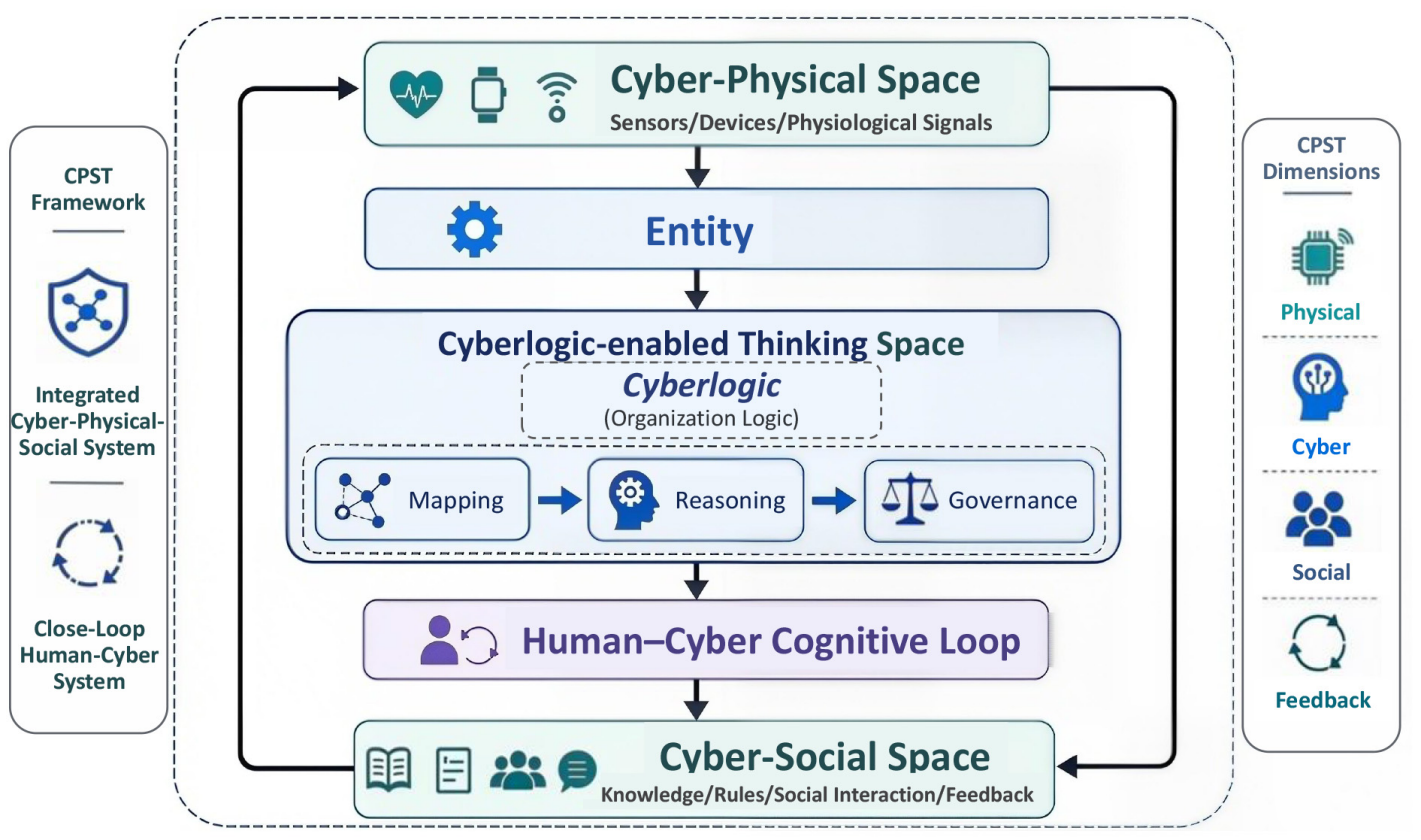
Cyberlogic-driven CyTS within the CPST framework. Cyberlogic provides the organizational logic that structures and orchestrates the cyberlogic-enabled thinking space (CyTS), linking physical inputs, cyber reasoning, and social feedback into a closed-loop human–cyber cognitive system.

### 3.2 Definition of Cyberlogic-enabled Thinking Space

Equation 1 defines CyTS through the following architectural tuple:


$$\begin{array}{l} CyTS=\langle E,M,R,G,H,C\rangle .1 \end{array}$$ (1)

This notation records components and their intended interactions; it does not constitute an algebra with proven closure or convergence properties. Equations 2–5 provide a minimal state-transition specification:


$$\begin{array}{l} S_{t}=M\left(E_{t}\right), \end{array}$$ (2)

$$\begin{array}{l} D_{t}=R\left(S_{t},K,U_{t};{\text{Π}}_{t}\right), \end{array}$$ (3)

$$\begin{array}{l} D_{t}^{\prime }=G\left(D_{t},HC_{t}|E_{x},R_{c},H_{j},{\text{Π}}_{t}\right), \end{array}$$ (4)

$$\begin{array}{l} S_{t+1}=F\left(S_{t},D_{t}^{\prime },F_{t}\right). \end{array}$$ (5)

Here, *E*_*t*_ comprises timestamped inputs; *S*_*t*_ is a structured state whose numerical encoding is implementation-dependent; *K* contains domain knowledge; and *U*_*t*_ records uncertainty. The policy specification Π_*t*_ constrains inference as well as action selection, ensuring that governance is not confined to an output filter. *D*_*t*_ contains candidate decisions and their evidence records, *HC*_*t*_ records human–cyber collaboration, and $D_{t}^{\prime }$ is an authorized decision or an abstention/escalation outcome. *E*_*x*_, *R*_*c*_, and *H*_*j*_ denote explainability, risk-control, and human-judgment constraints. Environmental feedback *F*_*t*_ supports a controlled transition F; safety and stability require separate verification.

This definition emphasizes three points. First, human thinking remains the cognitive subject. CyTS does not replace human cognition, but provides cyber-enabled cognitive support. Second, cyber cognitive models serve as augmentation components. Their role is to expand representation, reasoning, and decision-support capabilities, rather than to become autonomous cognitive agents. Third, human-cyber collaboration mechanisms provide the structural connection between human cognition and cyber cognition (Pariser, 2011). They enable bidirectional interaction, feedback updating, and governance regulation. Thus, CyTS is not equivalent to artificial consciousness or strong artificial intelligence. It is a cognitive augmentation structure that combines human cognitive agency with cyber computational capability under governance constraints. To improve formal consistency and engineering interpretability, the core mathematical symbols and functional variables used throughout the CyTS framework are summarized in Table 2.

**Table 2:** Mathematical symbols and functional definitions in CyTS.

Symbol	Definition	Functional meaning
*CyTS*	Cyberlogic-enabled Thinking Space	Governance-aware human–cyber cognitive architecture
*E*	Entity perception space	Multimodal cognitive input acquisition
*M*	Cognitive mapping function	Transformation from raw signals to structured representations
*R*	Multi-logic reasoning function	Hybrid reasoning and inference coordination
*G*	Governance feedback function	Governance-aware cognitive regulation
*H*	Human cognitive participation	Human judgment, supervision, and feedback
*C*	Cyber cognitive models	AI-based and cyber computational reasoning models
*S*	Structured cognitive state	Internal semantic representation at time *t*
*S*_*t*+1_	Updated cognitive system state	Adaptive cognitive state after feedback updating
*K*	Knowledge constraints	Knowledge graph and domain-specific constraints
*U*	Uncertainty states	Probabilistic uncertainty representation
*D*	Reasoning outputs	Intermediate inference and recommendation results
*D*′	Governance-regulated outputs	Final regulated decisions
*E*_*t*_	Multimodal entity inputs at time t	Dynamic perception inputs
*HC*	Human-cyber collaborative cognition	Hybrid human-AI cognitive state
*F*_*t*_	Environmental feedback	External adaptive feedback signals
α, β, γ, δ	Reasoning weights	Adaptive contribution coefficients
λ	Human-cyber collaboration coefficient	Relative cognitive authority allocation
ϕ_*e*_	Explainability governance weight	Governance emphasis on interpretability
ϕ_*r*_	Risk-control governance weight	Governance emphasis on safety
ϕ_*h*_	Human-judgment governance weight	Governance emphasis on human oversight
*E*_*x*_	Explainability constraints	Constraints for transparent reasoning
*R*_*c*_	Risk-control constraints	Safety and ethical regulation constraints
*H*_*j*_	Human judgment constraints	Human supervisory and intervention constraints

### 3.3 Cyberlogic-to-CyTS structural mapping

Cyberlogic provides the structural basis for implementing CyTS. The four Cyberlogic layers can be mapped into four functional layers of CyTS. Based on this theoretical position, the four Cyberlogic layers can be further mapped into the functional architecture of CyTS, as shown in Figure 2. The detailed mapping relationship between Cyberlogic and CyTS is summarized in Table 3.

**Figure 2:**
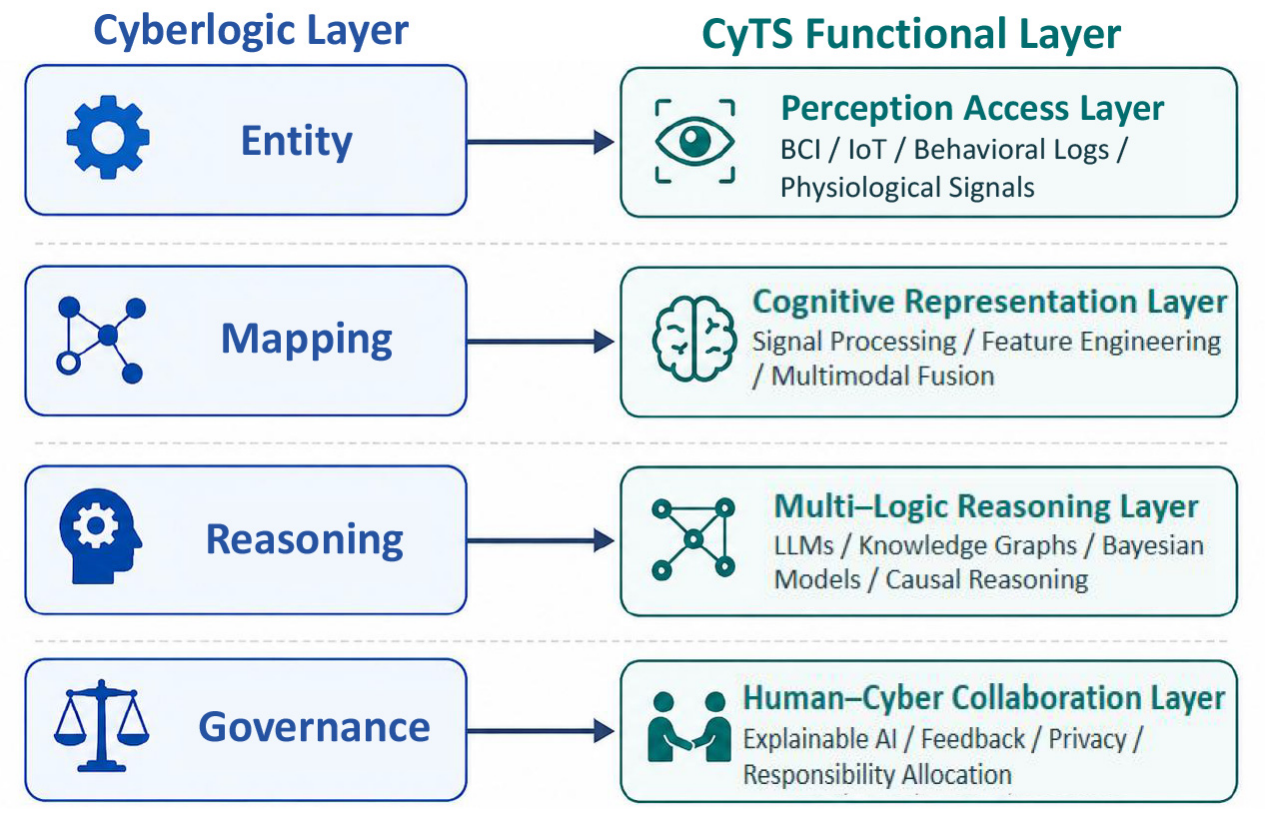
Cyberlogic-to-CyTS four-layer architecture. The cyberlogic layers of entity, mapping, reasoning, and governance are systematically mapped onto the perception access, cognitive representation, multi-logic reasoning, and human–cyber collaboration layers of CyTS, respectively, forming a governance-aware closed-loop human–cyber cognitive architecture.

**Table 3:** Cyberlogic-to-CyTS structural mapping.

Cyberlogic layer	CyTS functional layer	Core function	Representative technologies
Entity	Perception access layer	Acquisition and identification of cognition-related entities and signals	BCI, behavioral logs, IoT sensors, wearable devices
Mapping	Cognitive representation layer	Transformation of raw cognitive data into structured features and representations	Signal processing, feature engineering, multimodal fusion, embeddings
Reasoning	Multi-logic reasoning layer	Inference, prediction, explanation, and decision support	Large models, knowledge graphs, Bayesian models, causal reasoning
Governance	Human–cyber collaboration layer	Human feedback, responsibility allocation, ethical control, and adaptive updating	Explainable AI, human-in-the-loop interfaces, privacy protection, access control

This mapping transforms Cyberlogic from a theoretical framework into an implementable cognitive architecture. Unlike conventional AI pipelines, the Governance layer is not placed outside the system. Instead, it is embedded as a structural component that regulates reasoning outputs, feedback updating, and human–cyber collaboration (Rahwan et al., 2019; Dellermann et al., 2019).

### 3.4 CyTS within the CPST coupling structure

CyTS does not operate independently. It is dynamically coupled with cyber-enabled physical space and cyber-enabled social space.

Cyber-enabled Physical Space provides objective data from sensors, devices, environments, and biological signals. Cyber-enabled Social Space provides social knowledge, interaction records, collective experience, institutional rules, and domain norms (Amershi et al., 2019). CyTS integrates these inputs through cyber cognitive models and generates decision support for human cognition.

The CPST coupling process can be summarized as follows. Physical and social signals are first collected through the Entity layer. These signals are transformed into structured cognitive representations through the Mapping layer. The reasoning layer performs inference and generates recommendations. The Governance layer then regulates the output through human review, explainability, ethical constraints, and feedback mechanisms. The final decision influences physical actions and social interactions, thereby re-entering the CPST loop (Brundage et al., 2020; Ning et al., 2018).

### 3.5 Research hypotheses and validation criteria

The following hypotheses concern future implementations, not results of the present conceptual study. Comparisons should hold the underlying model, data access, action set, and task conditions constant where possible. All thresholds, primary outcomes, and minimally meaningful effects must be specified before evaluation.

**H1 (causal-logical transparency):** Compared with the same decision-support model using *post-hoc* explanations, CyTS increases the proportion of decisions with independently checkable evidence and assumption records. The traceability rate is defined in Equation 6 as follows:


$$\begin{array}{l} TR=N_{\text{traceable}}/N_{\text{total}},6 \end{array}$$ (6)

Record completeness must be assessed separately from correctness. Primary checks include validity of formal steps, explicit identification assumptions for intervention queries, and faithful correspondence between recorded steps and the executed workflow. SHAP values and perceived explanation quality are supplementary indicators, not causal-validity measures.

**H2 (decision quality):** CyTS improves task utility relative to both human-only and AI-only baselines under predefined uncertainty conditions, without exceeding a safety non-inferiority margin. Measure task success, severity-weighted errors, abstention, coverage, and error recovery; include the human time needed to review evidence.

**H3 (adaptation):** governance-controlled updating improves recovery after a defined distribution shift relative to a fixed-model baseline, without unacceptable oscillation, constraint violations, or rollback frequency. Specify the disturbance, observation horizon, and acceptable recovery region in advance. No convergence guarantee follows from the architecture alone.

**H4 (cross-space coordination):** explicit temporal and semantic contracts reduce inconsistent cross-space decisions while keeping end-to-end latency within the application's deadline. Measure unit and timestamp mismatches, synchronization failures, and deadline violations. Additional checks may increase latency, so consistency and timeliness are evaluated jointly.

**H5 (resource efficiency):** relative to an otherwise identical unconstrained generation workflow, selective reasoning and verification reduce energy per accepted correct decision at matched safety and coverage. Measure total pipeline energy, including retrieval, inference, verification, monitoring, and retries, alongside latency and human review time. A shorter explanation or fewer output tokens is not sufficient evidence of an energy saving.

Effect sizes and confidence intervals should be reported with prespecified acceptance margins, sampling units, and multiplicity controls. A nonsignificant superiority test is inconclusive rather than proof of equivalence or falsification. A claimed benefit is unsupported when its prespecified criterion is not met and contradicted when sufficiently precise estimates exclude the minimally meaningful benefit or reveal an unacceptable trade-off. Observational datasets such as MIMIC-IV may support retrospective workflow assessment, but cannot alone establish the effects of deploying an intervention. Causal tests require identified designs or simulations with known intervention ground truth.

## 4 Cyberlogic-driven cognitive architecture of CyTS

### 4.1 Overall architecture

The implementation of CyTS is not the deployment of a single AI algorithm. It is a Cyberlogic-driven cognitive architecture composed of perception access, cognitive representation, multi-logic reasoning, and human–cyber collaboration layers.

The architecture follows the principle that perception, reasoning, and governance should not be separated (Nguyen et al., 2014). Instead, they should be integrated into a closed-loop cognitive system. The system receives cognitive signals from humans, physical environments, and social contexts; transforms them into structured representations; conducts multi-logic reasoning; generates interpretable recommendations; receives human feedback; and updates models or constraints accordingly (Floridi et al., 2018).

Although CyTS is organized through four fundamental layers, the architecture is designed to support future scalability and extension. Adaptive evolution is considered a cross-layer capability rather than an independent Adaptation Layer. Through closed-loop feedback among Entity, Mapping, Reasoning, and Governance layers, CyTS can continuously adjust cognitive representations, reasoning strategies, and collaboration mechanisms according to environmental changes and human feedback. This design preserves the conceptual integrity of the Cyberlogic framework while enabling flexible adaptation in evolving CPST scenarios.

Furthermore, future implementations may introduce additional cross-layer interaction pathways for specific application requirements. For example, direct verification channels between the Entity and Governance layers can support privacy protection, security monitoring, and runtime compliance checking. Such extensions enhance architectural scalability without altering the fundamental Entity–Mapping–Reasoning–Governance organization.

### 4.2 Perception access layer

The perception access layer corresponds to the Entity layer of Cyberlogic. Its function is to identify and acquire cognition-related entities and signals. These signals may include brain signals, physiological indicators, behavioral logs, language inputs, environmental data, and social interaction records.

Brain-computer interfaces provide a direct or indirect channel between human cognitive states and cyber systems (Patrick-Krueger et al., 2025; Edelman et al., 2019). Non-invasive EEG-based BCIs can capture attention, fatigue, workload, or simple intention-related signals. However, current BCI technologies remain limited in spatial resolution, robustness, and semantic decoding capability. Therefore, within CyTS, BCI should be understood as an auxiliary cognitive input channel rather than a mind-reading technology (Lotte et al., 2013).

Behavioral data are also important cognitive inputs. Interaction logs, click sequences, writing behavior, browsing paths, response time, and task-switching patterns can reflect cognitive rhythm, decision strategies, and workload. Compared with neural signals, behavioral data are easier to collect and more suitable for long-term cognitive modeling.

### 4.3 Cognitive representation layer

The cognitive representation layer corresponds to the Mapping layer of Cyberlogic. Its function is to transform heterogeneous raw signals into structured cognitive representations (Moor et al., 2023; Huang et al., 2023).

Different data sources require different mapping strategies. EEG signals can be transformed into frequency-domain, time-frequency, and connectivity features. Behavioral logs can be transformed into temporal patterns, sequence features, and interaction profiles. Language inputs can be transformed into semantic embeddings, contextual vectors, and intent representations. Multimodal fusion can integrate these features into unified cognitive representations (Sundrani et al., 2023; Shi et al., 2025).

Each mapped object should carry a source identifier, timestamp, units, transformation history, and uncertainty descriptor. Relations must state whether they encode observed association, a domain rule, or an assumed causal mechanism. This prevents an embedding similarity or knowledge-graph edge from silently becoming an intervention claim. Mapping failures, incompatible units, and stale observations are explicit reasons to request new evidence or abstain.

### 4.4 Multi-logic reasoning layer

The multi-logic reasoning layer corresponds to the reasoning layer of cyberlogic. Its function is to integrate multiple reasoning paradigms to generate inference results, explanations, and decision recommendations. To conceptually illustrate the composition of the reasoning process, the reasoning outcome can be represented as:


$$\begin{array}{c} D_{t}=\alpha \cdot L_{\text{data}}\left(S_{t}\right)+\beta \cdot L_{\text{knowledge}}\left(S_{t},K\right)+\gamma \cdot L_{\text{uncertainty}}\left(S_{t},U_{t}\right)\\ +\delta \cdot L_{\text{human}}\left(S_{t},H\right)7 \end{array}$$ (7)

$$\begin{array}{l} \alpha +\beta +\gamma +\delta =18 \end{array}$$ (8)

The reasoning coefficients may be adapted to runtime context and normalized as aggregation weights. Entropy of an explicitly specified uncertainty distribution and semantic compatibility with domain knowledge are possible routing signals, but their suitability and scaling require empirical calibration. Normalization alone does not give these weights a causal or probabilistic interpretation. One possible normalization is:


$$\begin{array}{l} w_{i}=\frac{\exp\left({\tilde{w}}_{i}\right)}{\sum_{j\in \left\{\alpha ,\beta ,\gamma ,\delta \right\}}\exp\left({\tilde{w}}_{j}\right)},\;i\in \left\{\alpha ,\beta ,\gamma ,\delta \right\}9 \end{array}$$ (9)

Here, ${\tilde{w}}_{i}$ is a finite context-dependent score; normalization gives positive weights summing to one. It does not guarantee decision quality.

In practical implementations, the large models component within the reasoning layer is not limited to a specific model type. It may include general-purpose foundation models, such as large language models, as well as domain-adapted models customized for specific CPST applications. General-purpose models provide broad semantic understanding and reasoning capabilities, while domain-specific models, knowledge graphs, and specialized inference modules can be integrated to improve contextual accuracy and reliability in application scenarios such as healthcare and education. Therefore, the reasoning layer should be regarded as a heterogeneous reasoning framework that coordinates multiple models and knowledge sources rather than a direct deployment of a single large model.

Uncertainty logic supports probabilistic updating under incomplete information, while data-driven methods provide representation learning and predictive inference (Mnih et al., 2015; Geirhos et al., 2020). Knowledge-structured methods expose semantic relations and domain constraints; causal use additionally requires an explicit mechanism and intervention semantics. Human collaboration contributes contextual judgment and values. CyTS coordinates these roles while preserving the distinction between predictive, deductive, causal, and normative claims (Bender et al., 2021).

The weighted expression in Equations 7–9 is meaningful only when component outputs are mapped to comparable action scores with a declared calibration procedure. It must not add proofs, probabilities, and human judgments as though they were interchangeable quantities. The weights are routing or aggregation parameters, not evidence that the combined output is causally valid. Hard constraints and verified contradictions take precedence over weighted scores. In particular, knowledge-graph structure supplies causal information only when its edges have explicit causal semantics and supporting assumptions.

For every candidate, the Reasoning layer supplies an evidence record


$$A_{t}=\langle q,T,M,B,P,V,U_{t}\rangle ,$$

where *q* is the query, T its claim type (associative, deductive, interventional, counterfactual, or exploratory), M the model and version, B the assumptions, P the evidence and derivation provenance, and V the verification status. An LLM may propose a causal graph or translate a question, but an intervention claim must additionally identify its estimand and assumptions and pass an appropriate solver or domain check. A BDL/BQLA module would use the same contract while declaring its bipolar encoding and inference rules. Outputs from different formalisms remain distinguishable; unresolved disagreement triggers review rather than unqualified fusion.

### 4.5 Human–cyber collaboration layer

The human–cyber collaboration layer corresponds to the governance layer of cyberlogic. Its function is to regulate system outputs, support human decision-making, collect feedback, and maintain responsibility boundaries. The human-cyber collaborative outcome can be conceptually expressed as:


$$\begin{array}{c} HC=\lambda_{t}\cdot H+\left(1-\lambda_{t}\right)\cdot C,\\ \lambda_{t}=\max\left(0,\min\left(1,\lambda_{0}-\omega_{1}\cdot W\left(E_{t}\right)+R\left(U_{t}\right)\right)\right) \end{array}$$ (10)

In Equation 10, *H* and *C* must denote comparable decision-support scores if numerical aggregation is used. The coefficient λ_*t*_ describes assistance allocation, not a transfer of legal or professional responsibility. Workload *W*(*E*_*t*_), risk *R*(*U*_*t*_), and the scaling parameter ω_1_ require domain calibration; *R*(*U*_*t*_) here is a scalar risk index, distinct from the reasoning function *R*. Fatigue alone must not authorize increased autonomy. If a high-risk task exceeds the available human review capacity, the system defers, escalates to another qualified operator, or enters an independently specified fallback mode.

This mechanism enables flexible cognitive allocation between humans and cyber systems while preserving human oversight and responsibility. Through explainable interfaces and governance mechanisms, users can review, modify, or provide feedback on system recommendations. Such feedback contributes to the refinement of cognitive representations, reasoning strategies, and governance mechanisms within the closed-loop process.

This layer ensures that cognitive augmentation does not become cognitive replacement. Human agency, responsibility, and value judgment remain structurally embedded in the system.

## 5 Cyberlogic mechanisms for human–cyber cognitive integration

### 5.1 Organizational logic of cognitive coupling

The central function of Cyberlogic in CyTS is to organize cognitive coupling. Conventional AI systems often separate data acquisition, model inference, user interaction, and governance regulation. This separation can complicate feedback coordination, responsibility allocation, and adaptation.

Cyberlogic overcomes this limitation by integrating the four layers into one cognitive loop. An entity enables cognitive signals to enter the system. Mapping transforms them into structured representations. Reasoning generates inference through multi-logic coordination. Governance regulates outputs and introduces human feedback into the system. CyTS's Cyberlogic-driven operation is supported by multiple complementary logic mechanisms. As summarized in Table 4, these mechanisms are not independent modules, but are organized across the mapping, reasoning, and governance layers. Based on this multi-logic structure, the following subsections further analyze the uncertainty, data-driven, and human–cyber collaborative logics in CyTS.

**Table 4:** Multi-logic mechanisms in cyberlogic-driven CyTS.

Logic mechanism	Cyberlogic position	Main function	Typical methods	Role in CyTS
Uncertainty logic	Reasoning layer	Handles incomplete information, ambiguity, and dynamic risk	Bayesian inference, probabilistic graphical models, uncertainty quantification	Supports risk-aware and adaptive cognitive inference
Data-driven logic	Mapping and reasoning layers	Learns patterns and representations from multimodal data	Neural networks, large models, multimodal learning	Expands cognitive representation and prediction capability
Knowledge-structured logic	Reasoning layer	Provides explicit semantic relations and domain constraints	Knowledge graphs, ontologies, causal models	Improves interpretability and domain consistency
Human–cyber collaborative logic	Governance layer	Integrates human judgment, feedback, and value constraints	Human-in-the-loop, explainable AI, interactive decision support	Preserves human agency and responsibility
Governance logic	Governance layer	Regulates outputs, privacy, responsibility, and ethical constraints	Privacy protection, access control, audit trails, ethical governance	Ensures trustworthy and controllable cognitive augmentation

### 5.2 Uncertainty logic

Cognitive tasks often involve incomplete information, dynamic environments, and ambiguous evidence. Therefore, CyTS requires uncertainty logic to support probabilistic reasoning (Gao et al., 2021). In medical diagnosis, risk assessment, and dynamic decision-making, Bayesian inference and uncertainty quantification can support continuous belief updating in response to new evidence.

Within CPST, evidence may come from physical, social, and thinking spaces. Therefore, uncertainty logic enables CyTS to integrate heterogeneous information and continuously update beliefs in the presence of incomplete information.

### 5.3 Data-driven logic

Data-driven logic supports representation learning, prediction, and generation. Neural networks, large language models, and multimodal models can learn complex mappings from high-dimensional data and generate useful cognitive representations (Willett et al., 2023; Baltrušaitis et al., 2019).

Within CyTS, data-driven logic expands cognitive representation capability (Ngiam et al., 2011; Shrestha et al., 2022). It enables the system to identify latent patterns in behavioral logs, physiological signals, medical images, educational records, and traffic data. However, data-driven logic is primarily based on statistical correlations and may suffer from limited interpretability, hallucination, bias, and weak causal reasoning (Chang and Bergen, 2024; Gilbert et al., 2024). Therefore, data-driven logic cannot serve as the only logic of CyTS. It must be combined with uncertainty logic, knowledge-structured logic, and human–cyber collaborative logic.

### 5.4 Human–cyber collaborative logic

Human–cyber collaborative logic reflects the core feature of CyTS. The system does not make decisions independently, nor does it reduce humans to passive validators. Instead, humans and cyber models participate in a structured cognitive loop (Collins et al., 2024). Human feedback establishes a cycle of:


$$\begin{array}{c} \text{Input}\to \text{Representation}\to \text{Reasoning}\to \text{Human Review}\to \\ \text{Governance Feedback}\to \text{Model Update} \end{array}$$

Through this loop, CyTS can continuously adapt to users, tasks, and environments while maintaining human control (Shneiderman, 2020).

### 5.5 Governance-centric cognitive feedback

Governance is the distinguishing feature of Cyberlogic-driven CyTS. In conventional AI systems, governance is often external to model reasoning. In CyTS, governance constraints directly regulate reasoning outputs. Governance feedback can be represented as:


$$\begin{array}{c} D_{t}^{\prime }=\arg\underset{d\in \Omega \left(R_{c}\right)}{\max}[\phi_{e}\cdot f_{\exp}\left(d|E_{x}\right)+\phi_{h}\cdot f_{\text{human}}\left(d|H_{j}\right)\\ -\phi_{r}\cdot f_{\text{risk}}\left(d|R_{c}\right)],\;\sum \phi_{i}=1 \end{array}$$ (11)

The set Ω(*R*_*c*_) contains actions admissible under the implemented constraints; membership is not an unconditional guarantee of real-world safety. The scores in Equation 11 require explicit scales and validation. In particular, *f*_exp_ should assess evidence coverage, declared assumptions, and verified reasoning status; feature attribution alone cannot certify causality. For actions requiring causal justification, the associated record $A_{t}$ must pass the relevant identification and verification checks. If the admissible set is empty, evidence is inadequate, or a deadline is exceeded, the system abstains or invokes a domain-validated fallback and records the reason. The fallback itself must have an operating envelope and an accountable owner.

The governance formulation specifies how interpretability requirements, risk policies, and human review can regulate candidate decisions (Tonekaboni et al., 2019). Its effectiveness depends on the correctness of the implemented constraints, the quality of evidence, and the capacity of reviewers. Governance can block known violations and expose unsupported assumptions; it cannot guarantee that all harmful outcomes are anticipated.

### 5.6 Governance operationalization mechanisms

While governance is often discussed as a high-level principle in human–AI systems, practical deployment requires governance to be implemented through executable mechanisms that directly influence system behavior. Therefore, the Governance layer of CyTS is operationalized through five complementary governance components: Privacy Governance, Accountability Governance, Human Oversight Governance, Responsibility Allocation Governance, and Runtime Monitoring Governance.

- **Privacy governance**: privacy Governance regulates the collection, storage, access, and utilization of cognitive data throughout the CyTS lifecycle. Since CyTS may process sensitive information such as behavioral records, physiological signals, cognitive states, and decision preferences, privacy protection cannot be treated as an external compliance requirement (Kosinski, 2017). Instead, privacy constraints participate directly in data access and reasoning processes through mechanisms such as access control, anonymization, differential privacy, and secure data management.
- **Accountability governance**: accountability Governance ensures that reasoning processes remain traceable and auditable. Each recommendation generated by CyTS is associated with supporting evidence, reasoning pathways, governance interventions, and human feedback records. This traceability allows system outputs to be reviewed, explained, and audited when disputes or failures occur. Consequently, accountability is embedded within the cognitive loop rather than being evaluated only after decision execution.
- **Human oversight governance**: human Oversight Governance preserves meaningful human control over system behavior. Human participants may review, modify, reject, or override system-generated recommendations according to contextual requirements and domain regulations. Unlike conventional human-in-the-loop systems where intervention is limited to specific stages, oversight in CyTS remains available throughout the entire cognitive cycle. This mechanism prevents excessive automation and helps maintain human agency in safety-critical scenarios.
- **Responsibility allocation governance**: responsibility Allocation Governance defines the distribution of responsibilities among developers, deployers, institutions, intelligent agents, and end users. Because decisions emerge through human–machine collaboration, responsibility cannot be assigned solely to either humans or AI systems. CyTS therefore adopts traceable responsibility allocation mechanisms that clarify decision ownership and accountability across different operational roles.
- **Runtime monitoring governance**: runtime Monitoring Governance continuously evaluates system behavior during operation. It detects abnormal outputs, policy violations, unsafe recommendations, model drift, and unexpected interactions. When governance violations are identified, corrective actions may be triggered automatically or escalated to human supervisors. This mechanism enables governance to function as a continuous regulatory process rather than a static rule set.

Implementation requires concrete privacy, audit, and monitoring policies. Where differential privacy is used, the mechanism, adjacency relation, privacy budget ε_*DP*_, and composition accounting must be stated; access control remains a separate requirement. Each audit record links the decision identifier and timestamp to $A_{t}$, the governance action, human feedback, final decision, and model/policy version. Risk above a prespecified threshold triggers escalation or a validated fallback. Threshold selection, missed detections, and false alarms must be evaluated within the application operating envelope.

Collectively, these governance mechanisms transform governance from a conceptual principle into an executable component of cognition. Governance therefore participates directly in reasoning, decision generation, feedback incorporation, and adaptive updating, forming a governance-aware cognitive loop within CyTS.

## 6 Closed-loop operation and representative applications

### 6.1 Closed-loop cognitive operation process

The Cyberlogic-driven CyTS system operates as a closed-loop cognitive process rather than a linear pipeline (Morey et al., 2025). The operational process includes multi-source cognitive input, cognitive representation, multi-logic reasoning, human–cyber collaborative decision-making, governance feedback, and adaptive updating.

CyTS first collects data from physical, social, cyber, and human cognitive contexts. These data include sensor readings, physiological signals, EEG signals, behavioral logs, language inputs, social rules, and domain knowledge. The system then transforms heterogeneous data into structured cognitive representations through signal processing, feature extraction, semantic embedding, and multimodal fusion.

The system performs reasoning by integrating uncertainty logic, data-driven logic, knowledge-structured logic, and human–cyber collaborative logic. System outputs are presented to human users through interpretable interfaces, and human users evaluate recommendations based on professional knowledge, contextual judgment, and value considerations. Feedback is used to update model parameters, knowledge structures, interface strategies, or governance rules.

The update rule is specified as a controlled transition rather than an unspecified semantic gradient:


$$\begin{array}{l} S_{t+1}=\{\begin{array}{ll} F_{\theta_{t}}\left(S_{t},D_{t}^{\prime },F_{t}\right), & \text{if the update passes validation},\\ S_{t}, & \text{otherwise}, \end{array} \end{array}$$ (12)

where θ_*t*_ identifies the approved model and policy configuration. A rejected model update does not suspend input monitoring: new observations are remapped at the next cycle. Parameter changes require versioning, regression checks, authorization, and a rollback plan. No universal learning-rate interval or stability theorem is assumed. In physical applications, stability must be assessed against the relevant process dynamics and control constraints, rather than inferred from small changes in an internal representation.

Unlike conventional linear AI pipelines, CyTS operates as a recursive cognitive loop. The closed-loop operational process is shown in Figure 3. Conventional systems usually stop after output generation. By contrast, CyTS continuously re-enters the cognitive loop through human evaluation, environmental feedback, and governance adjustment. This recursive mechanism enables long-term co-adaptation between humans and cyber systems. To further illustrate the implementability of CyTS, the iterative human-cyber cognition can be represented as a simplified algorithmic workflow. Algorithm 1 summarizes the major operational steps from entity perception to governance-guided adaptation.

**Figure 3:**
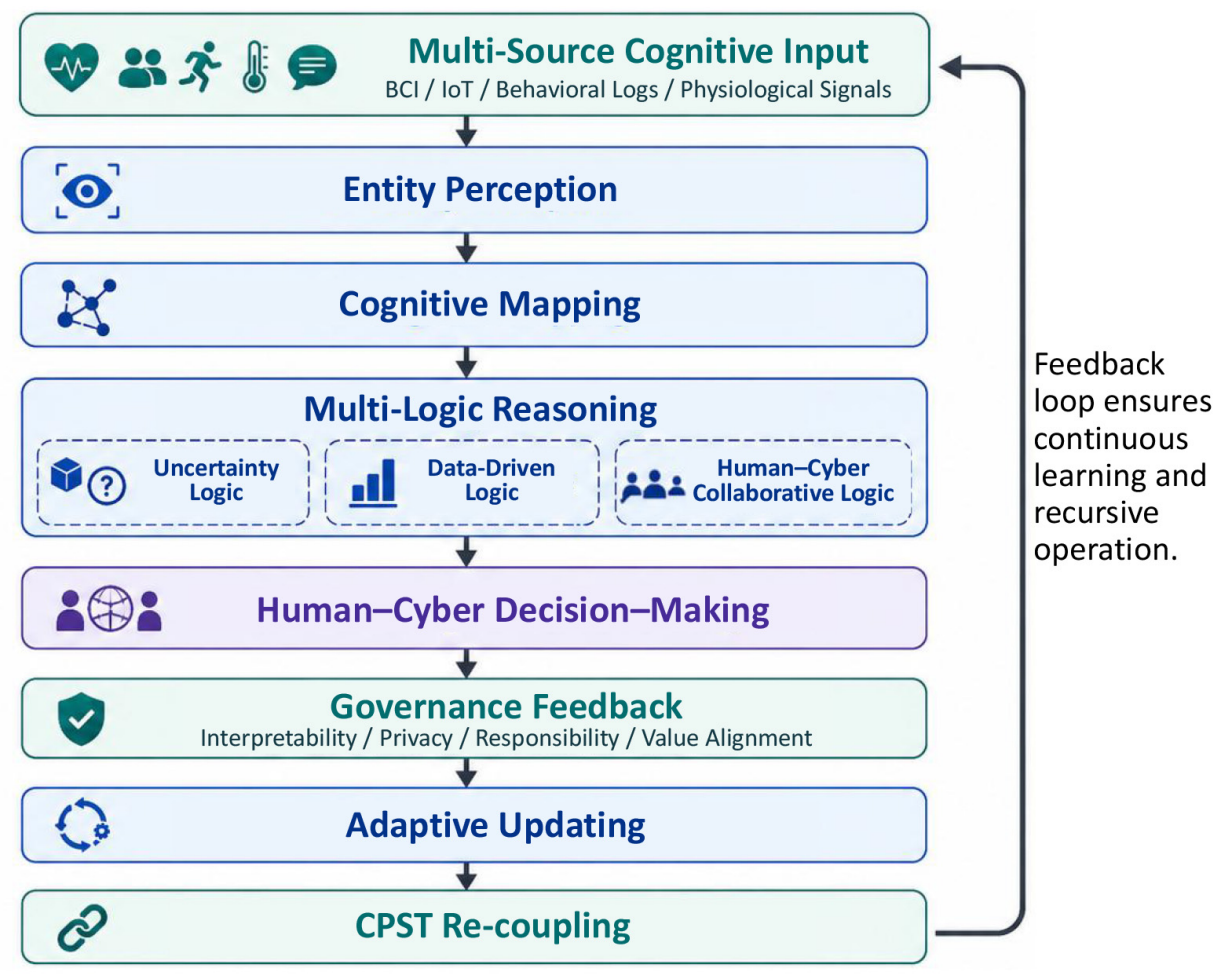
Closed-loop operation of CyTS.

Algorithm 1 Evidence-aware, governance-controlled CyTS cycle. 
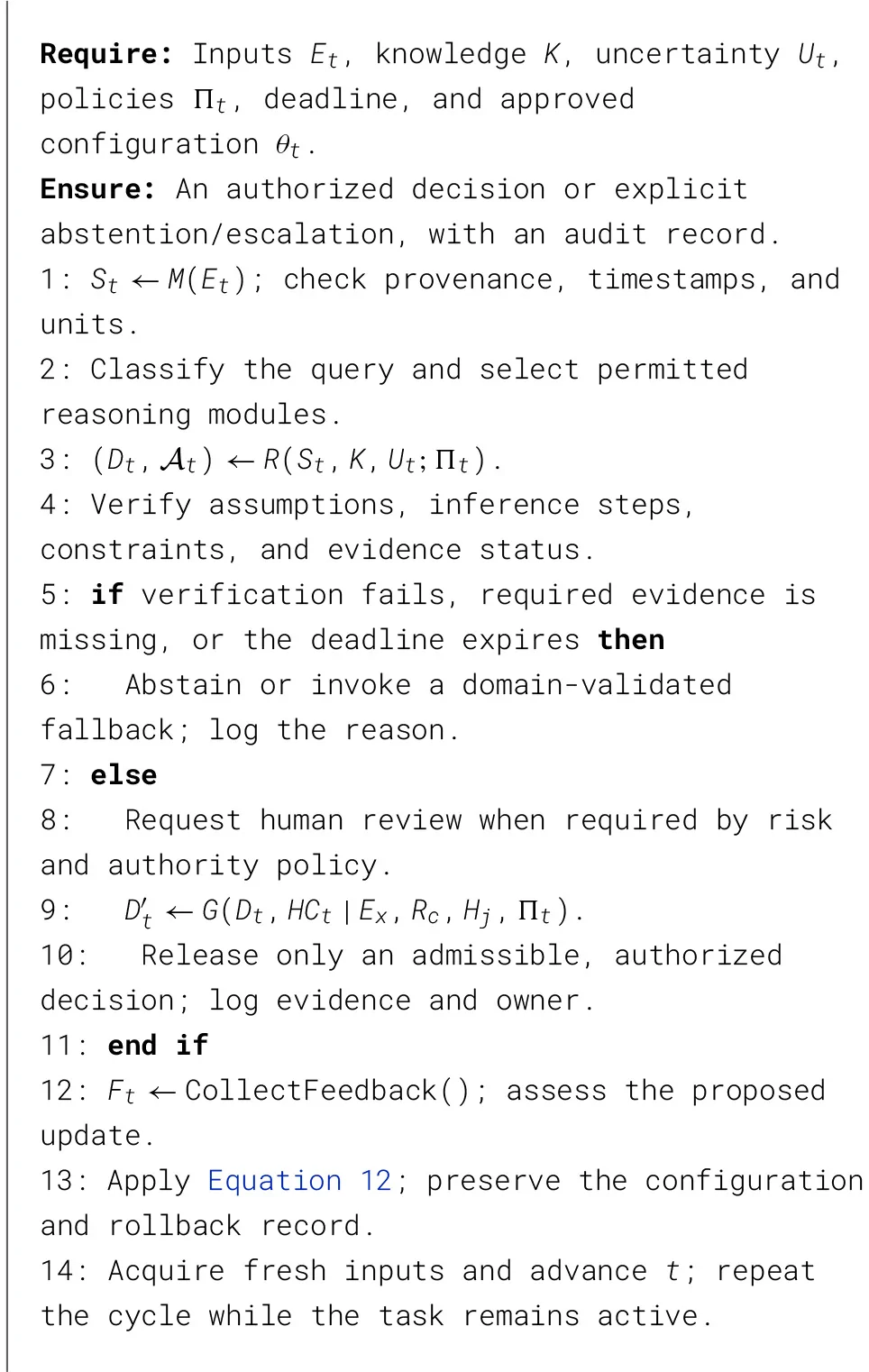

The operational process also reflects the coupling structure of CPST. Physical space contributes objective environmental data; social space contributes collective knowledge and institutional rules; cyber space contributes computational modeling capability; and thinking space contributes human cognition, interpretation, and judgment. CyTS coordinates these spaces through cyberlogic-driven organization.

Another key feature is the coexistence of machine-speed processing and human-speed cognition. Cyber models can process multimodal information rapidly and continuously, while humans provide contextual understanding, ethical reasoning, and strategic judgment. CyTS, therefore, does not attempt to eliminate human participation. Instead, it distributes cognitive functions between humans and cyber systems according to their complementary strengths.

The adaptive updating stage is especially important for long-term system evolution. Human feedback can refine knowledge structures, modify reasoning priorities, adjust governance rules, and personalize interaction mechanisms. Over time, CyTS gradually forms domain-specific cognitive adaptation capabilities.

From a systems perspective, CyTS can therefore be understood as a governance-aware cognitive infrastructure rather than an isolated AI model. Its value lies not only in prediction capability but also in continuous cognitive coordination, feedback integration, and organizational stability.

### 6.2 Cross-domain architectural mapping templates

Although healthcare, education, and transportation differ significantly in operational objectives, environmental constraints, and data modalities, representative CyTS-oriented architectural mappings across these domains share a common Cyberlogic-driven organizational structure. As shown in Table 5 (Ning et al., 2016), CyTS organizes heterogeneous human–cyber systems through a unified closed-loop architecture consisting of entity, mapping, reasoning, governance, and feedback layers. Table 6 summarizes the functional responsibilities and data exchange relationships among the core components.

**Table 5:** Unified CyTS structure across application domains.

Dimension	Intelligent healthcare	Smart education	Intelligent transportation
Entity input	Physiological signals, medical images, EEG, wearable data	Learning behavior, eye movement, keyboard logs, classroom interaction	Road sensors, GPS, vehicle data, video streams, weather data
Mapping	Multimodal medical feature extraction	Learning state representation and knowledge tracing	Traffic state representation and risk feature extraction
Reasoning	Diagnostic models, disease risk prediction, medical knowledge graphs	Learning analytics, recommendation models, student state prediction	Traffic prediction, route planning, congestion analysis
Governance	Physician review, clinical responsibility, privacy protection	Teacher intervention, educational fairness, learner privacy	Dispatcher control, safety regulation, responsibility allocation
Feedback	Clinician correction and outcome validation	Teacher feedback and learner performance	Driver response and traffic control outcomes
Core value	Diagnostic reliability and workload reduction	Personalized learning and timely intervention	Safety, efficiency, and congestion reduction

**Table 6:** Evidence and control contracts at CyTS interfaces.

Interface	Required payload	Failure response
Entity–mapping	Source, timestamp, units, consent/access status, missingness	Reject unauthorized input; flag missing or stale evidence.
Mapping–reasoning	Versioned representation, transformation provenance, uncertainty, relation semantics	Resolve incompatible scales or meanings before inference.
Reasoning–governance	Candidates and $A_{t}$: claim type, model, assumptions, provenance, verification status	Withhold unsupported causal claims; expose conflicts and identification failure.
Governance–action	Authorized action or abstention, responsible actor, constraints, deadline, fallback	Escalate or defer if approval, timing, or admissibility checks fail.
Feedback–update	Outcome, action/exposure history, model/policy version, validation and rollback record	Quarantine unvalidated updates; preserve prior approved configuration.

### 6.3 Applications of CyTS in different domains

While Table 5 illustrates the shared organizational structure of CyTS across domains, different application scenarios impose distinct governance requirements, human-oversight mechanisms, and operational constraints. Accordingly, CyTS is presented not as a domain-specific AI system with empirically validated performance, but as a conceptual generalized governance-aware human–cyber cognitive architecture adaptable to heterogeneous CPST environments.

Intelligent healthcare represents the most safety-critical CyTS scenario because medical environments require high levels of interpretability, accountability, and human supervision (Chen et al., 2022). Existing AI-assisted diagnostic systems, multimodal clinical decision-support platforms, and medical imaging systems increasingly rely on collaborative human–AI cognition. Within the CyTS perspective, these systems can be conceptually interpreted as governance-aware cognitive augmentation structures in which physicians remain responsible for reviewing recommendations, validating explanations, overriding automated outputs, and ensuring clinical accountability.

As shown in Figure 4, CyTS organizes medical human–AI collaboration as a recursive governance-aware cognitive loop integrating multimodal perception, structured reasoning, physician supervision (Wolpaw and Wolpaw, 2012; Lebedev and Nicolelis, 2006), and adaptive feedback (Tetsuka et al., 2024; Oganesian and Shanechi, 2024). Rather than replacing physician cognition, the architecture supports evidence-based reasoning and reduces information-processing burden while preserving human-centered medical authority.

**Figure 4:**
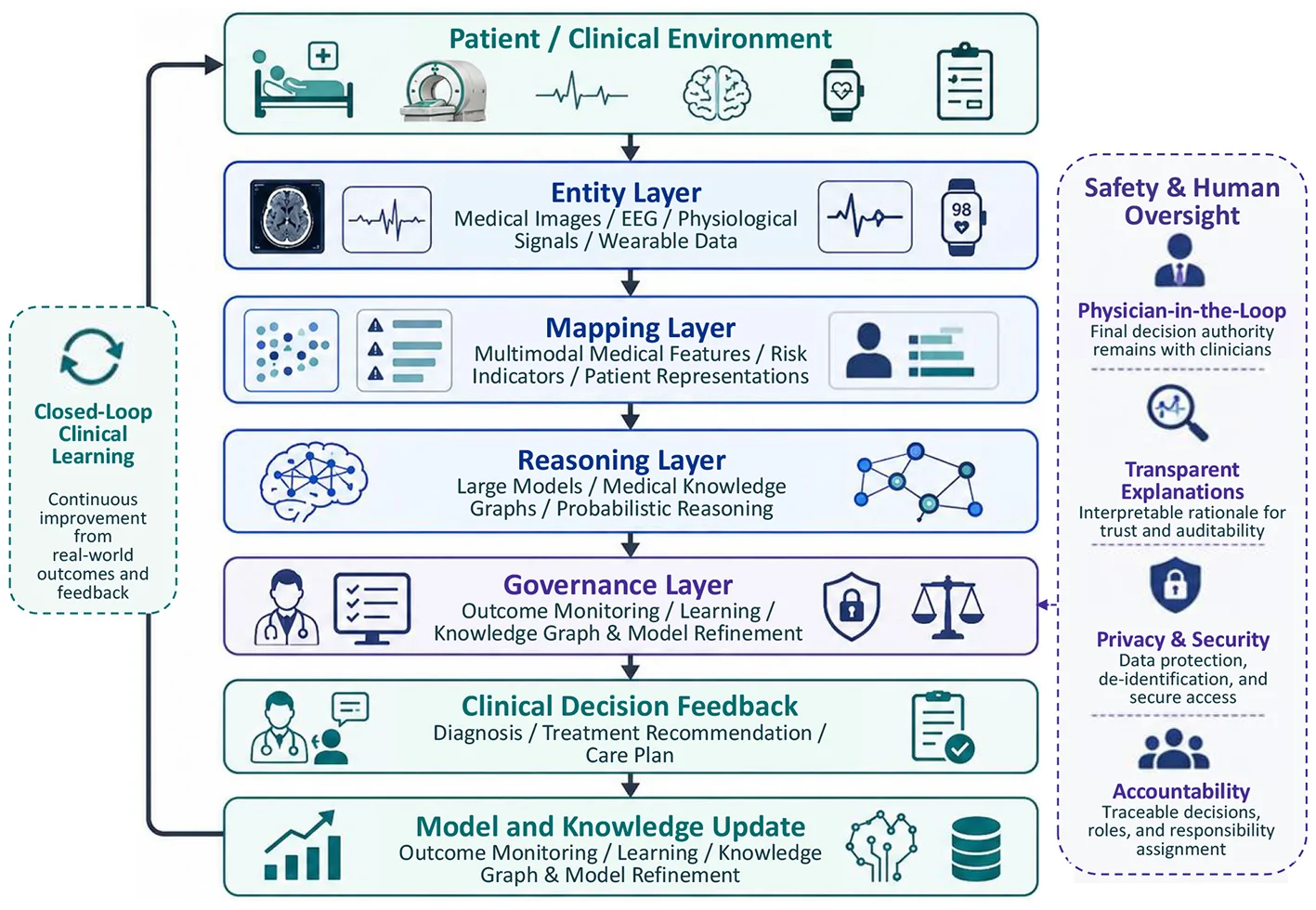
Cyberlogic-driven CyTS operation in intelligent healthcare.

In smart education, the primary challenge lies in balancing adaptive personalization with fairness, pedagogical responsibility, and learner autonomy. Although recommendation algorithms and learning analytics may offer potential benefits for individualized instruction, educational governance remains fundamentally teacher-centered. Under the CyTS framework, teachers maintain supervisory authority over algorithmic recommendations and contextual educational decisions, thereby preventing purely automated optimization from dominating pedagogical processes.

In intelligent transportation, governance challenges are closely associated with safety regulation, real-time coordination, and responsibility allocation during high-risk operational situations. Existing intelligent traffic-management infrastructures combine predictive AI systems with human dispatching mechanisms to support traffic control and route optimization. Human operators remain responsible for supervisory intervention and safety-critical decision coordination, ensuring that adaptive traffic-management strategies can be designed to remain accountable and controllable under dynamic real-world conditions.

The causal distinction changes the workflow in each domain. In healthcare, a risk prediction is not evidence that a proposed treatment improves outcomes: the record must state the treatment, endpoint, population, confounding assumptions, and available clinical evidence, with clinician review before action. In education, a correlation between a learning resource and achievement does not establish an intervention benefit; evaluation must account for prior attainment and resource assignment. In transportation, an accurate congestion forecast does not establish that signal retiming is safe: candidate actions require a validated dynamics model or controlled simulation and hard traffic constraints. These examples specify requirements rather than report application outcomes.

For a concrete transportation test, let queue length evolve as *x*_*t*+1_ = max{0, *x*_*t*_+*a*_*t*_−*s*(*x*_*t*_, *u*_*t*_)}, where arrivals *a*_*t*_, signal action *u*_*t*_, and discharge function *s* are explicitly defined. The system records which parts of *s* are measured, assumed, or calibrated; simulates competing actions under arrival uncertainty; checks pedestrian and conflict constraints; and logs the selected action and outcome. A queue equilibrium requires matched arrival and discharge rates under suitable conditions, not merely a repeated observational pattern. This classical testbed can compare alternative relational or equilibrium modules without assuming quantum causation.

Despite domain-specific differences, these scenarios share the same underlying CyTS organizational characteristics: multimodal perception, structured cognitive mapping, multi-logic reasoning coordination, governance-aware human oversight, and recursive feedback adaptation. This structural consistency suggests that Cyberlogic can serve as an organizational foundation for human–cyber cognitive augmentation across heterogeneous CPST scenarios.

## 7 Evaluation framework and governance challenges

While the foregoing applications are conceptual illustrations, practical deployment of CyTS in real-world settings would require systematic evaluation. Since CyTS directly influences human cognition and decision-making, its evaluation should not be limited to model accuracy alone (Burton et al., 2024). Instead, CyTS should be regarded as a governance-aware human–cyber cognitive system integrating reasoning quality, interpretability, collaboration effectiveness, adaptability, and preservation of human agency (Devlin et al., 2019). To improve operational applicability, each evaluation dimension should include measurable indicators and corresponding assessment methods. Table 7 summarizes a governance-aware evaluation framework for CyTS systems.

**Table 7:** Evaluation dimensions for CyTS systems.

Evaluation dimension	Core question	Representative indicators	Measurement protocol/instrument
Cognitive augmentation	Does the system improve human cognitive performance?	Task accuracy, completion time, cognitive workload reduction	Human-only vs. CyTS-assisted comparison; NASA-TLX
Collaborative gain	Does human–cyber collaboration outperform either side alone?	Error correction rate, collaborative efficiency	Human-only vs. AI-only vs. human–cyber comparison
Interpretability	Can users understand system outputs and reasoning processes?	Explanation usefulness, uncertainty awareness	SHAP/LIME explanation analysis; user trust evaluation
Governance effectiveness	Are risks and ethical constraints effectively controlled?	Auditability, bias monitoring, privacy compliance	Governance audit and fairness evaluation
Adaptability	Can the system improve through feedback?	Feedback utilization rate, update stability	Longitudinal adaptive-performance tracking
Human agency preservation	Does the system preserve human decision authority?	Override frequency, automation-bias reduction	Human intervention and override analysis

### 7.1 Causal and resource evaluation protocol

A first prototype should compare a common predictive/LLM backbone in four conditions: baseline assistance, governance logging only, governance plus explicit causal verification, and an exploratory equilibrium module where an executable implementation exists. Keep data, hardware, model versions, prompts, and action constraints fixed, and use paired task instances with repeated runs. Human-facing comparisons require controlled assignment and a prespecified analysis of workload and automation bias. Simulator interventions with known ground truth and formal causal benchmarks complement observational evaluations; neither replaces prospective domain validation.

For intervention queries, score correct identification or justified non-identification, effect-estimation error when ground truth exists, and sensitivity to hidden-confounder or mechanism-shift scenarios. Independently audit whether reported evidence corresponds to executed reasoning. Include failures and abstentions in the denominator when reporting coverage, so that selective refusal cannot create an artificial gain in reliability. Equilibrium modules must additionally state solution existence, multiplicity, stability conditions, and behavior away from equilibrium.

Measure whole-pipeline energy per completed task and per accepted correct decision, alongside the coverage and safety results. State the measurement boundary, hardware utilization, batch size, output length, idle-power treatment, and repeated-run uncertainty. Report latency and human effort separately. The aim is to determine whether selective verification avoids enough unnecessary computation to offset its own overhead; CyTS currently provides no measured energy advantage.

### 7.2 Evidence boundaries and research agenda

CyTS currently offers an organizational specification, not a validated cognitive theory or a deployed causal engine. The proposed interfaces improve auditability only if implementations faithfully record computation and independently check evidence. They do not resolve all causal identification problems, guarantee privacy, or prove closed-loop stability. Causal validity, normative acceptability, and human authorization are separate conditions: an effective intervention may still be inadmissible.

Equilibrium-based and EMTI-inspired components remain optional research extensions. Before they influence deployed actions, they should provide formal semantics, reproducible implementations, measurable predictions, and comparisons with classical alternatives under the same resources. A claim involving physical quantum effects additionally requires an operationally specified physical system and suitable measurements. Architectural compatibility or a network analogy alone cannot meet that requirement. The near-term priority is a conventional prototype that tests the evidence contracts and governance gates; broader foundational claims remain open to separate investigation.

However, achieving satisfactory performance across these dimensions remains challenging in real-world deployments. In particular, interpretability, human agency preservation, privacy protection, and responsibility allocation constitute the most critical governance concerns affecting trustworthy human–cyber cognitive augmentation systems. The major governance challenges of CyTS can be summarized as follows:

1. **Interpretability, trust, and transparency:** since CyTS directly influences human cognition and decision-making, users must understand not only system recommendations but also the supporting evidence, reasoning process, and associated uncertainty. Consequently, explainability should be embedded within the Governance layer rather than treated as a *post-hoc* add-on (Bubeck et al., 2023; Ji et al., 2023; Hogan et al., 2021; Rudin, 2019).
2. **Human agency preservation and automation bias:** as CyTS systems become increasingly capable, users may become overly dependent on automated recommendations, resulting in reduced professional judgment and automation bias (Silver et al., 2016). Maintaining meaningful human control therefore becomes a fundamental governance requirement. CyTS addresses this issue through uncertainty visualization, counterfactual explanations, decision confirmation mechanisms, and human override capabilities that preserve human agency throughout the cognitive loop.
3. **Privacy and cognitive data protection:** CyTS processes highly sensitive information, including cognitive states, physiological signals, behavioral patterns, and decision preferences. Governance-aware privacy mechanisms such as anonymization, differential privacy (Dwork, 2006), access control, and secure data management are therefore essential. Privacy protection should be integrated directly into the system architecture rather than imposed as an external compliance mechanism.
4. **Responsibility allocation and accountable governance:** responsibility allocation becomes increasingly complex because decisions emerge from interactions between human cognition and cyber recommendations (Topol, 2019; Kamar, 2016; Esteva et al., 2017). Inspired by contemporary AI governance principles and the notion of meaningful human control, CyTS incorporates human-in-the-loop supervision, decision traceability, risk-tier governance, and human override mechanisms. Under this structure, developers, deployers, institutions, and end users assume differentiated yet traceable responsibilities, while humans retain final authority in safety-critical situations.

## 8 Conclusion

This paper proposed CyTS as a governance-centric organizational architecture for human–cyber cognition in CPST environments. Its entity–mapping–reasoning–governance structure connects heterogeneous representations, reasoning evidence, human judgment, and controlled adaptation. The framework distinguishes logical validity and predictive association from intervention-relevant causal support, and specifies evidence records, component interfaces, and decision gates that preserve these distinctions. Healthcare, education, and transportation scenarios illustrate how those requirements affect decisions; they do not demonstrate effectiveness or deployment readiness.

The theoretical scope includes structural causal models and equilibrium-based causal perspectives, while brain–universe analogies and EMTI are situated as exploratory directions with distinct evidentiary requirements. CyTS does not claim that entity modeling, symbolic inference, or a proposed quantum account is individually sufficient for cognition. Future work should implement the specified contracts, test transparency and decision-quality hypotheses under controlled interventions and distribution shifts, and measure energy and latency at matched safety and coverage. Any claimed benefit of a new causal formalism must be established through reproducible comparisons rather than inferred from architectural integration.

## Data Availability

The original contributions presented in the study are included in the article/supplementary material, further inquiries can be directed to the corresponding authors.
